# New Toolset of Reporters Reveals That Glycogen Granules Are Neutral Substrates of Bulk Autophagy in *Komagataella phaffii*

**DOI:** 10.3390/ijms252111772

**Published:** 2024-11-01

**Authors:** Nimna V. Wijewantha, Praneetha Battu, Kuangcai Chen, Ravinder Kumar, Taras Y. Nazarko

**Affiliations:** 1 Department of Biology, Georgia State University, Atlanta, GA 30303, USA; 2 Imaging Core Facility, Georgia State University, Atlanta, GA 30303, USA; kchen@gsu.edu; 3 Section of Molecular Biology, Division of Biological Sciences, University of California San Diego, La Jolla, CA 92093, USA; fravinde@uthsc.edu

**Keywords:** autophagy, bulk autophagy, CBM20, glycogen, glycogen granules, glycophagy, *Komagataella phaffii*, *Pichia pastoris*, selective autophagy, yeast

## Abstract

Glycogen, a branched polysaccharide organized into glycogen granules (GGs), is delivered from the cytoplasm to the lysosomes of hepatocytes by STBD1-driven selective autophagy (glycophagy). Recently, we developed *Komagataella phaffii* yeast as a simple model of GG autophagy and found that it proceeds non-selectively under nitrogen starvation conditions. However, another group, using *Saccharomyces cerevisiae* as a model, found that glycogen is a non-preferred cargo of nitrogen starvation-induced bulk autophagy. To clarify cargo characteristics of *K. phaffii* GGs, we used the same glycogen synthase-based reporter (Gsy1-GFP) of GG autophagy in *K. phaffii* as was used in *S. cerevisiae*. The *K. phaffii* Gsy1-GFP marked the GGs and reported on their autophagic degradation during nitrogen starvation, as expected. However, unlike in *S. cerevisiae*, glycogen synthase-marked GGs were delivered to the vacuole and degraded there with the same efficiency as a cytosolic glycogen synthase in glycogen-deficient cells, suggesting that glycogen is a neutral cargo of bulk autophagy in *K. phaffii*. We verified our findings with a new set of reporters based on the glycogen-binding CBM20 domain of human STBD1. The GFP-CBM20 and mCherry-CBM20 fusion proteins tagged GGs, reported about the autophagy of GGs, and confirmed that GGs in *K. phaffii* are neither preferred nor non-preferred substrates of bulk autophagy. They are its neutral substrates.

## 1. Introduction

Glycogen is a branched storage polysaccharide in bacteria, yeast, and animals, including humans. It is associated with several enzymes and regulatory proteins forming the glycogen–protein complex known as a glycogen granule (GG) [1]. The protein glycogenin initiates glycogen synthesis by generating a short-chain glucose oligomer covalently bound to itself. Then, the glycogenin-bound oligosaccharide serves as a substrate for glycogen synthase, which adds more glucose units to the linear glycogen chain. The glycogen branching enzyme contributes multiple branching points to linear chains creating a large complex structure of the GG [2,3,4]. In many organisms, glycogen biosynthesis occurs to cope with starvation conditions, which eventually lead to cytoplasmic degradation of glycogen by glycogen phosphorylase and glycogen debranching enzyme, and autophagic degradation of GGs inside lysosomes/vacuoles by acid α-glucosidase [5,6].

Glycogen metabolism is highly regulated, and malfunctioning of this process is the main cause of many glycogen storage diseases, such as Pompe disease, which is characterized by the accumulation of GGs inside lysosomes due to proficiency in autophagy and deficiency in the lysosomal acid α-glucosidase [7,8]. Autophagy/macroautophagy is a membrane trafficking pathway that delivers cytoplasmic constituents (either selectively or non-selectively) to animal/human lysosomes or yeast/plant vacuoles in the double-membrane vesicles called autophagosomes [9,10,11]. Autophagy is the only known route by which cytosolic GGs can reach the lytic compartment from yeast to mammals [7,8,12,13]. Therefore, it is important to know the type (selective or non-selective) and mechanism of GG autophagy, which can help to discover new therapeutic targets for Pompe disease.

There is still a controversy as to what type of autophagy is responsible for the degradation of GGs in mammals. It was proposed to be selective and called “glycophagy”, because it is mediated by STBD1 (starch binding domain 1) protein that can act as a bridge between the glycogen and the Atg8-family proteins of the autophagic membrane in cultured mammalian cells [14,15]. However, further studies in a mouse model showed that the function of STBD1 in glycogen transport to lysosomes is tissue specific. While STBD1 plays a major role in delivering GGs to lysosomes in the liver, it is dispensable for this process in skeletal and cardiac muscles [16,17]. The autophagy of GGs in muscles might still be selective (as in the case of neonatal glycogenolysis [7]), just mediated by another bridging protein. However, it might also be less efficient (i.e., non-selective) compared to the liver. Recently, we introduced *Komagataella phaffii* (formerly *Pichia pastoris*) yeast as a new model of GG autophagy and showed that its efficiency in nitrogen-starved cells is comparable with the efficiency of non-selective autophagy [18,19] making the latter possibility of non-selective autophagy of GGs in non-hepatic mammalian tissues even more likely.

Interestingly, a recent study in *S. cerevisiae* found that GGs are spared from degradation by bulk autophagy during the first 24 h of nitrogen starvation [20]. However, prolonged starvation overrides this GG exclusion mechanism via the expression of the Atg45 protein, which bridges the glycogen and the Atg8-containing autophagic membrane. Therefore, in *S. cerevisiae*, glycogen is never degraded non-selectively (as a neutral cargo). It is either excluded from degradation by autophagy (as a non-preferred cargo) or selected for degradation by autophagy (as a preferred cargo) [20]. This is in sharp contrast to what we observed with glycogenin (Glg1-GFP) as a marker of GGs in *K. phaffii* [18]. Here, by using the same glycogen synthase-based reporter (Gsy1-GFP) for measuring glycogen autophagy as was used in *S. cerevisiae* [20], and by designing a new toolset of STBD1-based reporters, we established that glycogen in *K. phaffii* is neither a preferred nor a non-preferred autophagic cargo. Instead, it is a neutral cargo of bulk autophagy.

## 2. Results

### 2.1. K. phaffii Gsy1-GFP Marks GGs

**Figure 1:**
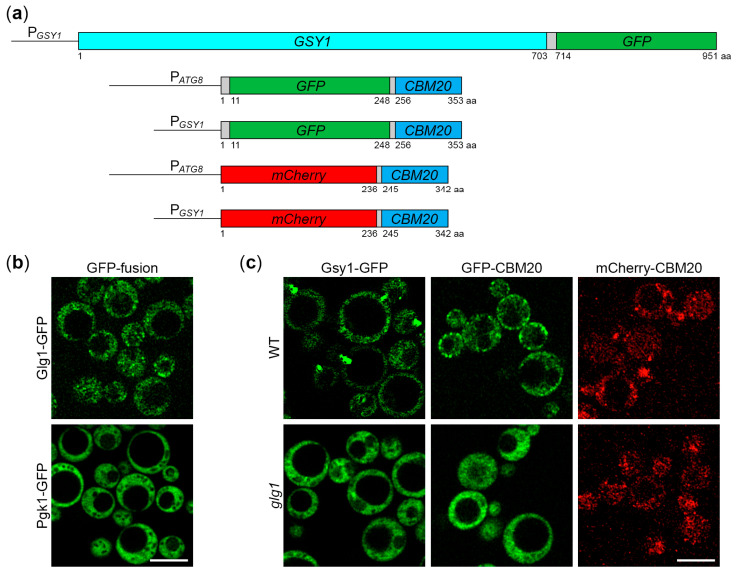
New marker proteins for glycogen granules (GGs) in *K. phaffii*. (**a**) Cassettes for the expression of Gsy1- and CBM20-based markers of GGs in *K. phaffii*. Gsy1-GFP fusion is expressed from the *GSY1* promoter, whereas GFP-CBM20 and mCherry-CBM20 fusions can be expressed from either *ATG8* or *GSY1* promoter. (**b**) Confocal microscopy can distinguish the localization patterns of GG (Glg1-GFP) and cytosolic (Pgk1-GFP) markers in *K. phaffii*. (**c**) Gsy1-GFP, GFP-CBM20, and mCherry-CBM20 fusions mark GGs. The localization patterns of fusion proteins expressed from the *GSY1* promoter were compared in wild-type (WT) and *glg1* strains with and without GGs, respectively. (**b**,**c**) Cells were grown in YPD for 1 day. Scale bar, 5 μm.

Recently, we developed *K. phaffii* as a simple model for studying the autophagy of GGs [18]. Using a reporter, which is covalently bound to GGs (Glg1-GFP), we showed that they are degraded by a non-selective process during nitrogen starvation for 24 h. However, another group, using Gsy2-GFP as a marker of GGs in *S. cerevisiae*, found that GGs are mostly spared from bulk autophagy during the first 24 h of nitrogen starvation [20]. The difference in results between the two studies could be due to a species specificity of the nitrogen starvation response or because different reporters (i.e., covalently bound Glg1-GFP versus non-covalently bound Gsy2-GFP) were used to measure glycogen autophagy. To exclude the latter, we decided to measure the autophagy of GGs in *K. phaffii* using the glycogen synthase-based reporter, as in *S. cerevisiae*. In *S. cerevisiae*, there are two genes of glycogen synthase, *GSY1* and *GSY2*, which appear after whole genome duplication. In *K. phaffii*, there is only one glycogen synthase, called Gsy1 (Figure S1). Therefore, we created a Gsy1-GFP fusion protein. For this, we generated the integrative plasmid, pRK23, with the P*_GSY1_*-*GSY1-GFP* expression cassette (Figure 1a).

To test if Gsy1-GFP fusion correctly localizes to GGs, we used confocal microscopy that previously allowed to distinguish the uneven distribution of the Gsy2-GFP reporter from the uniform cytosolic distribution of Pgk1-GFP fusion in *S. cerevisiae* [20]. First, we compared the localization patterns of established markers of GGs and cytosol in *K. phaffii*, Glg1-GFP, and Pgk1-GFP [18] (Figure 1b). Both fusion proteins were expressed from the same *GLG1* promoter to make their expression levels more comparable, as before [18]. In contrast to the diffuse cytosolic distribution of Pgk1-GFP, Glg1-GFP had a granular pattern of localization consistent with its covalent binding to GGs (Figure 1b). These results validated this method of GG visualization in *K. phaffii*. Then, we compared the localization of Gsy1-GFP in wild-type (WT) and *glg1* cells with and without GGs, respectively (Figure 1c). In WT cells, Gsy1-GFP had a granular pattern of localization, whereas *glg1* cells had a diffuse cytosolic pattern suggesting that Gsy1-GFP fusion is associated with GGs.

### 2.2. Gsy1-GFP Reports About the Autophagy of GGs

**Figure 2:**
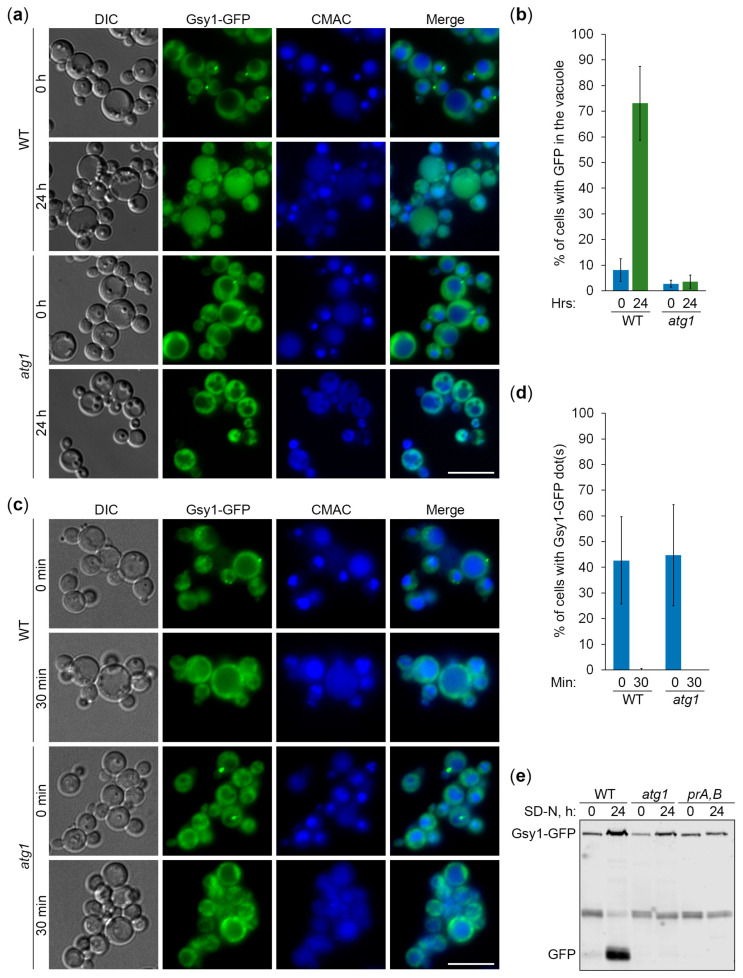
Gsy1-GFP reports about glycogen autophagy. (**a**) Delivery of Gsy1-GFP to the vacuole. WT and *atg1* cells with Gsy1-GFP were grown in YPD for 1 day. A portion of cells was transferred to SD-N for 24 h. Remaining YPD cultures were stained with CMAC for 30 min and imaged as “0 h”. The last 30 min of SD-N cultures was incubation with CMAC before imaging them as “24 h”. Scale bar, 10 µm. (**b**) Quantification of images in (**a**). Displayed are averages and standard deviations. (**c**) Dissipation of Gsy1-GFP dots before its delivery to the vacuole. The same cells were grown in YPD for 1 day. A fraction of cells was transferred to SD-N with CMAC for 30 min and imaged as “30 min” of nitrogen starvation. The rest of the YPD cultures were stained with CMAC for 30 min and imaged as “0 min” of nitrogen starvation. Scale bar, 10 µm. (**d**) Quantification of images in (**c**). Displayed are averages and standard deviations. (**e**) Processing of Gsy1-GFP in the vacuole. WT, *atg1*, and *prA,B* cells with Gsy1-GFP were grown in YPD for 1 day. A portion of the cells was transferred to SD-N. At 0 and 24 h, equal volumes of cultures (not equal biomass) were taken from SD-N for immunoblotting.

Previously, we showed that *K. phaffii* GGs are degraded by non-selective (bulk) autophagy in the first 24 h of nitrogen starvation [18]. Since Gsy1-GFP marks GGs, its delivery to the vacuole is expected to be dependent on autophagic machinery. To test this, we compared the vacuolar delivery of Gsy1-GFP in WT and *atg1* (autophagy related 1 [21]) cells by epifluorescence microscopy (Figure 2a), as we did before for Glg1-GFP [18]. Due to the lower resolution of epifluorescence microscopy compared to confocal microscopy, the granular pattern of Glg1-GFP by confocal microscopy (Figure 1b) presented itself as a diffuse cytosolic pattern by epifluorescence microscopy [18] after 0 h of nitrogen starvation. Similarly, Gsy1-GFP appeared mostly as diffuse cytosolic in WT and *atg1* strains at 0 h of nitrogen starvation (Figure 2a). However, in contrast to Glg1-GFP [18], Gsy1-GFP also localized to the dot-like structure in both strains. This dot-like structure disappeared after 24 h of nitrogen starvation in both WT and *atg1* cells, suggesting a non-autophagic mechanism of its removal. Importantly, the bulk of Gsy1-GFP was redistributed from the cytosol to the CMAC-stained vacuole in WT, but not *atg1*, cells at the 24 h time-point (Figure 2a,b), proving the autophagic delivery of Gsy1-GFP to the vacuole, as expected.

To exclude the possibility that the dot-like structure might decrease the Gsy1-GFP capacity to report about glycogen autophagy, we tested how quickly it dissipated under nitrogen starvation conditions. In our previous study, we encountered similar dot-like structures with the mutated Glg1^Y212F^-GFP fusion protein, but these structures fully dissolved in the first 30 min of nitrogen starvation before any significant delivery of Glg1^Y212F^-GFP to the vacuole [18]. Therefore, we imaged WT and *atg1* cells with Gsy1-GFP at 0 and 30 min points (Figure 2c) and counted the number of cells with Gsy1-GFP dot(s) (Figure 2d). Remarkably, the Gsy1-GFP dots disappeared in both WT and *atg1* cells by 30 min of nitrogen starvation suggesting that their dissipation is not only autophagy-independent, but also precedes bulk autophagy. As such, Gsy1-GFP dot-like structures in YPD medium do not interfere with the ability of Gsy1-GFP fusion to report about autophagic delivery of GGs to the vacuole in the medium without nitrogen (SD-N).

Following the delivery of *K. phaffii* GGs to the vacuole, GG-bound proteins, like Glg1-GFP, are degraded by vacuolar proteases leaving a proteolytically stable GFP moiety intact [18]. Since Gsy1-GFP marks GGs and is delivered to the vacuole by autophagy, its vacuolar processing is expected to be dependent on autophagic machinery and vacuolar proteolysis. To test this, we compared the processing of Gsy1-GFP in WT, *atg1*, and *pep4 prb1* (deficient in proteinases A and B [22]; called *prA,B* hereafter) cells by immunoblotting with GFP antibodies (Figure 2e), as we did before for Glg1-GFP processing [18]. We found that Gsy1-GFP is processed to GFP in WT cells, but not in *atg1* or *prA,B* cells, confirming that Gsy1-GFP undergoes autophagic degradation in the vacuole. As such, Gsy1-GFP can be used as an alternative reporter to monitor the autophagy of GGs in *K. phaffii*.

### 2.3. Gsy1-GFP-Marked Glycogen Is a Neutral Cargo of Bulk Autophagy in K. phaffii

In *S. cerevisiae*, glycogen was proposed to be a non-preferred cargo of bulk autophagy, i.e., a cargo, which is largely spared from degradation in the first 24 h of nitrogen starvation [20]. Indeed, if the *S. cerevisiae* Gsy2-GFP reporter binds GGs, it is barely processed to GFP after 24 h in SD-N. However, if Gsy2-GFP does not bind GGs (in glycogen-deficient cells), its processing to GFP increases several-fold [20]. We did not observe such an effect in *K. phaffii* when we compared the processing of GG-bound Glg1-GFP and cytosolic Glg1^Y212F^-GFP reporters [18]. To test it further with the *K. phaffii* counterpart of *S. cerevisiae* Gsy2, we performed the following experiments.

**Figure 3:**
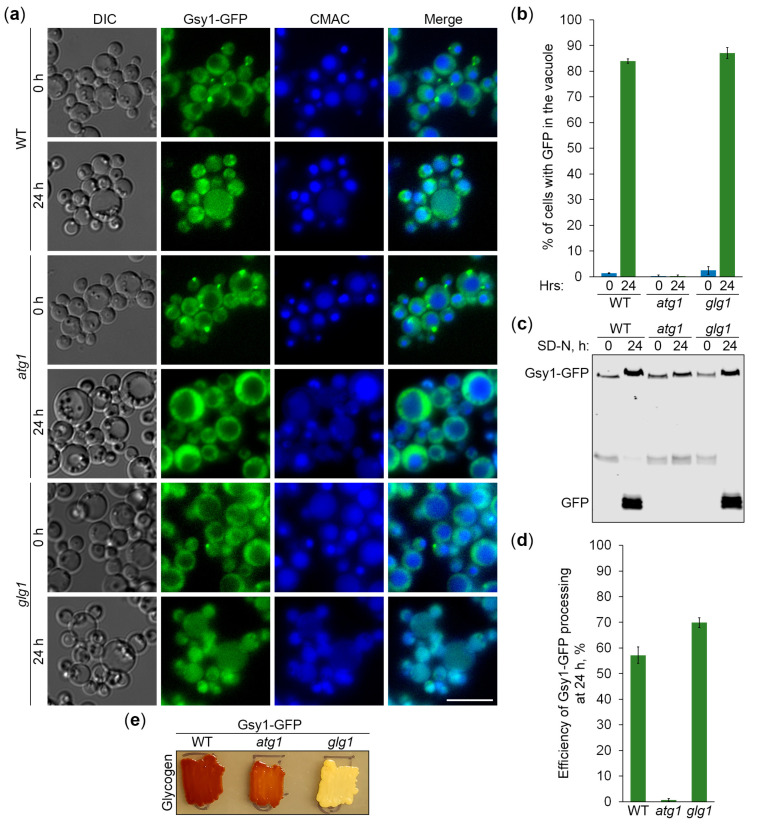
Gsy1-GFP-marked glycogen is a neutral autophagic cargo in *K. phaffii*. (**a**) Delivery of Gsy1-GFP to the vacuole. WT, *atg1*, and *glg1* cells with Gsy1-GFP were grown in YPD for 1 day. A fraction of the cells was transferred to SD-N for 24 h. The rest of the YPD cultures were stained with CMAC for 30 min and imaged as “0 h”. The last 30 min of SD-N cultures was incubation with CMAC before imaging them as “24 h”. Scale bar, 10 µm. (**b**) Quantification of images in (**a**). Displayed are averages and standard deviations. (**c**) Processing of Gsy1-GFP in the vacuole. The same cells were grown in YPD for 1 day. A fraction of the cells was transferred to SD-N. At 0 and 24 h, equal volumes of cultures (not equal biomass) were taken from SD-N for immunoblotting. (**d**) Quantification of immunoblotting in (**c**). Displayed are averages and standard deviations. (**e**) Glycogen content. The same cells were grown on YPD plate for 2 days and exposed to the vapor of iodine crystals for glycogen staining.

First, we studied the delivery of *K. phaffii* Gsy1-GFP to the vacuole in glycogen-proficient (WT) and glycogen-deficient (*glg1*) cells using *atg1* cells as a negative control (Figure 3a). Quantification of epifluorescence microscopy results showed that the percentage of cells with GFP signal in the vacuole at the 24 h point of nitrogen starvation neither increased nor decreased in *glg1* cells compared to WT cells (Figure 3b). An increase would be consistent with the glycogen being a non-preferred cargo of bulk autophagy, whereas a decrease would support the glycogen being a preferred cargo of autophagy. Instead, the localization of Gsy1-GFP to either GGs (WT) or the cytosol (*glg1*) produced the same vacuolar trafficking result, suggesting that GGs, similar to the cytosol, are a neutral cargo of bulk autophagy in *K. phaffii*.

To corroborate the above vacuolar delivery findings with vacuolar degradation data, we tested the Gsy1-GFP processing in the same set of strains by immunoblotting (Figure 3c). The immunoblotting identified a small (13%) difference in the processing of Gsy1-GFP to GFP between WT and *glg1* strains at 24 h of nitrogen starvation (Figure 3d), which is negligible compared to the several-fold difference in *S. cerevisiae* [20], supporting our conclusion that glycogen is a neutral cargo of bulk autophagy in *K. phaffii*. As an additional control, we compared the glycogen content in all the strains used in the delivery and degradation assays (Figure 3e). As expected, both WT and *atg1* strains with Gsy1-GFP synthesized glycogen, whereas *glg1* cells with Gsy1-GFP did not. This solidified our conclusion that there is neither exclusion of GGs nor selectivity for them during the nitrogen starvation-induced autophagy in *K. phaffii*. Therefore, *K. phaffii* GGs are a neutral cargo.

### 2.4. CBM20 Fusion Proteins Mark GGs in K. phaffii

To verify our findings with Gsy1-GFP, we used the CBM20 (carbohydrate-binding module, family 20) domain of the human STBD1 (starch binding domain 1) protein. Mammalian STBD1 acts as the autophagic receptor of GGs, tagging them for autophagic degradation via its C-terminal CBM20 domain that binds glycogen [14,15]. Recently, the CBM20 domain of human STBD1 was successfully used to detect GGs as a part of GYSC, the glycogen-binding probe containing the glutathione S-transferase, myc-tag, and CBM20 domain [23]. However, GYSC had to be expressed in bacteria, purified, and used in combination with primary and secondary antibodies to detect GGs in mammalian cells and tissues. This limited its use to ELISA and immunofluorescence microscopy [23]. To overcome these limitations, we created genetically encoded CBM20-based markers of GGs suitable for live microscopy and immunoblotting: GFP-CBM20 and mCherry-CBM20 (Figure 1a). For flexibility in expression levels, both fusions were placed separately under a weaker *ATG8* promoter and a stronger *GSY1* promoter. In summary, we created four integrative plasmids, pRK29, pRK34, pRK28, and pRK32, with P*_ATG8_*-*GFP-CBM20*, P*_GSY1_*-*GFP-CBM20*, P*_ATG8_*-*mCherry-CBM20*, and P*_GSY1_*-*mCherry-CBM20* expression cassettes, respectively (Figure 1a).

To check if GFP-CBM20 and mCherry-CBM20 fusions localize to GGs in *K. phaffii* cells, we used confocal microscopy, as with Gsy1-GFP (Figure 1c). Precisely, we compared the localization of CBM20 fusions in the WT strain with GGs and *glg1* mutant without GGs. In WT cells, GFP-CBM20 had a granular pattern of distribution, similar to Glg1-GFP and Gsy1-GFP, whereas in *glg1* cells, the distribution of GFP-CBM20 was diffuse cytosolic, resembling the distributions of Pgk1-GFP in WT cells and Gsy1-GFP in *glg1* cells (Figure 1b,c). Because the brightness of mCherry was much lower than the brightness of GFP, the cytosolic distribution of mCherry-CBM20 in *glg1* cells had a fine granular pattern. However, in WT cells, in addition to this fine granular background, it was easy to spot bigger granules (of mCherry-CBM20) that were common across all the GG markers (Figure 1b,c). As such, GFP-CBM20 and mCherry-CBM20, similar to Glg1-GFP and Gsy1-GFP, localized to GGs in glycogen-proficient cells of *K. phaffii* and could be used as their markers.

### 2.5. CBM20 Fusions Report About the Autophagy of GGs

**Figure 4:**
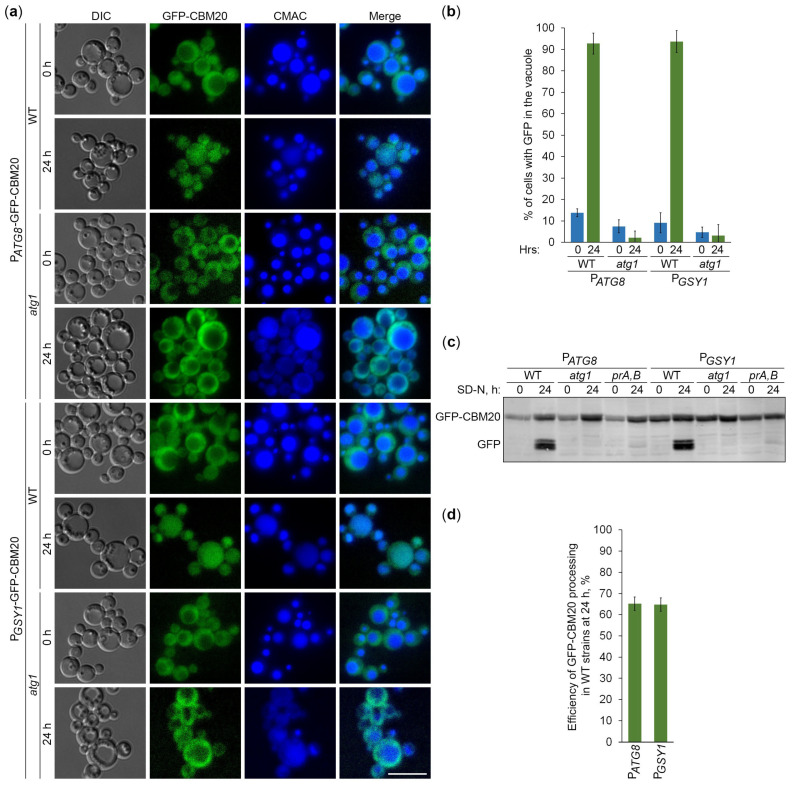
GFP-CBM20 reports about glycogen autophagy. (**a**) Delivery of GFP-CBM20 to the vacuole. WT and *atg1* cells expressing GFP-CBM20 from the corresponding promoter were grown in YPD for 1 day. A portion of the cells was transferred to SD-N for 24 h. Remaining YPD cultures were stained with CMAC for 30 min and imaged as “0 h”. The last 30 min of SD-N cultures was incubation with CMAC before imaging them as “24 h”. Scale bar, 10 µm. (**b**) Quantification of images in (**a**). Displayed are averages and standard deviations. (**c**) Processing of GFP-CBM20 in the vacuole. WT, *atg1*, and *prA,B* cells with GFP-CBM20 under corresponding promoters were grown in YPD for 1 day. A portion of the cells was transferred to SD-N. At 0 and 24 h, equal volumes of cultures (not equal biomass) were taken from SD-N for immunoblotting. (**d**) Quantification of immunoblotting in (**c**). Displayed are averages and standard deviations.

**Figure 5:**
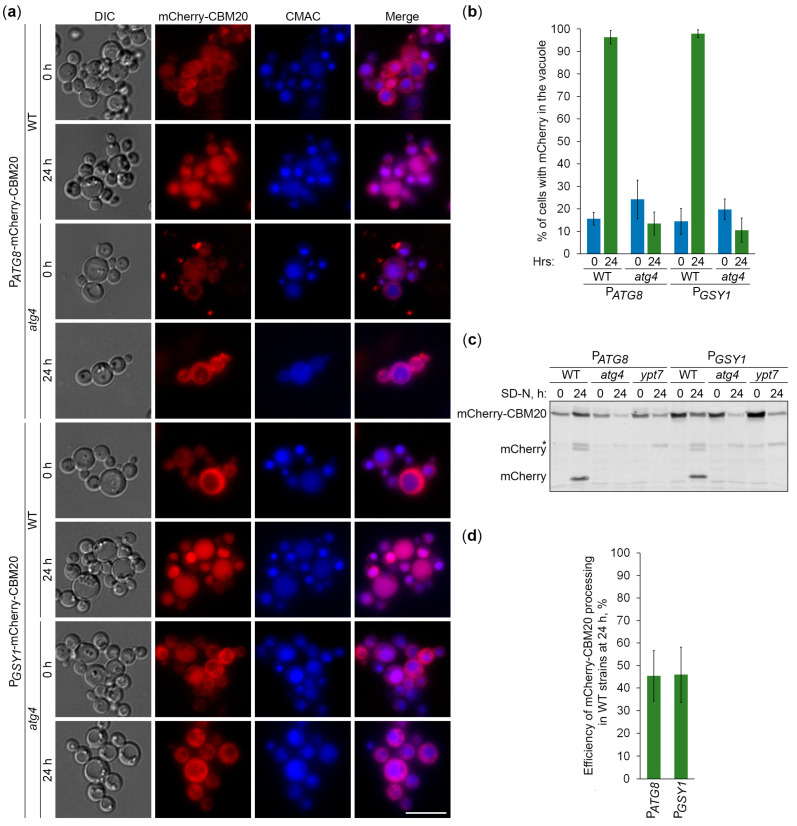
mCherry-CBM20 reports about glycogen autophagy. (**a**) Delivery of mCherry-CBM20 to the vacuole. WT and *atg4* cells expressing mCherry-CBM20 from the corresponding promoter were grown in YPD for 1 day. A portion of the cells was transferred to SD-N for 24 h. Remaining YPD cultures were stained with CMAC for 30 min and imaged as “0 h”. The last 30 min of SD-N cultures was incubation with CMAC before imaging them as “24 h”. Scale bar, 10 µm. (**b**) Quantification of images in (**a**). Displayed are averages and standard deviations. (**c**) Processing of mCherry-CBM20 in the vacuole. WT, *atg4*, and *ypt7* cells with mCherry-CBM20 under corresponding promoters were grown in YPD for 1 day. A portion of the cells was transferred to SD-N. At 0 and 24 h, equal volumes of cultures (not equal biomass) were taken from SD-N for immunoblotting. *, non-autophagic band. (**d**) Quantification of immunoblotting in (**c**). Displayed are averages and standard deviations.

Since CBM20 fusions mark GGs, their delivery to the vacuole is expected to be dependent on the core autophagic machinery. To verify this, we followed the vacuolar delivery of differentially expressed GFP-CBM20 in WT and *atg1* cells (Figure 4a) and differentially expressed mCherry-CBM20 in WT and *atg4* (autophagy related 4 [24]) cells (Figure 5a) by epifluorescence microscopy. Due to the lower resolution of epifluorescence microscopy compared to confocal microscopy, the granular localization of CBM20 fusions by confocal microscopy (WT in Figure 1c) appeared as diffuse cytosolic by epifluorescence microscopy in all the strains after 0 h of nitrogen starvation (Figure 4a or Figure 5a). Importantly, the bulk of CBM20 fusions was redistributed from the cytosol to the CMAC-stained vacuole in the WT strain, but not in *atg1* (Figure 4a) and *atg4* (Figure 5a) mutants after 24 h of nitrogen starvation. Interestingly, the percentage of WT cells with either GFP (Figure 4b) or mCherry (Figure 5b) signals in the vacuole was nearly identical for CBM20 fusions expressed from the *ATG8* and *GSY1* promoters. Together, these microscopy experiments proved the autophagic delivery of CBM20 fusions to the vacuole, as expected.

After the delivery of GGs to the vacuole, GG-associated proteins are expected to be degraded by vacuolar proteases, leaving proteolytically stable GFP and mCherry fragments intact. Since CBM20 fusions are delivered to the vacuole by autophagy, their vacuolar processing to GFP and mCherry is expected to be dependent on the core autophagic machinery, autophagosome-vacuole fusion, and vacuolar proteolysis. To verify this, we monitored processing of the differentially expressed GFP-CBM20 in WT, *atg1*, and *prA,B* cells (Figure 4c) and processing of the differentially expressed mCherry-CBM20 in WT, *atg4*, and *ypt7* (deficient in autophagosome-vacuole fusion [25]) cells (Figure 5c) by immunoblotting with GFP and mCherry antibodies, respectively. We found that CBM20 fusions are processed to GFP/mCherry fragments in WT cells, but not in *atg1, atg4*, *ypt7*, or *prA,B* cells. Even though the expression of CBM20 fusions from the *ATG8* promoter was lower than that from the *GSY1* promoter at the 0 h point, the expression of fusions from the two promoters was comparable at the 24 h point. Moreover, the efficiency of GFP-CBM20 (Figure 4d) and mCherry-CBM20 (Figure 5d) processing at the 24 h point in WT cells with P*_ATG8_* and P*_GSY1_* constructs was nearly the same. Altogether, these biochemical experiments indicate that CBM20 fusions undergo autophagic degradation in the vacuole. Therefore, they can be used as reporters to monitor the autophagy of GGs in *K. phaffii*.

### 2.6. Glycogen Marked with CBM20 Fusions Is a Neutral Cargo of Bulk Autophagy in K. phaffii

**Figure 6:**
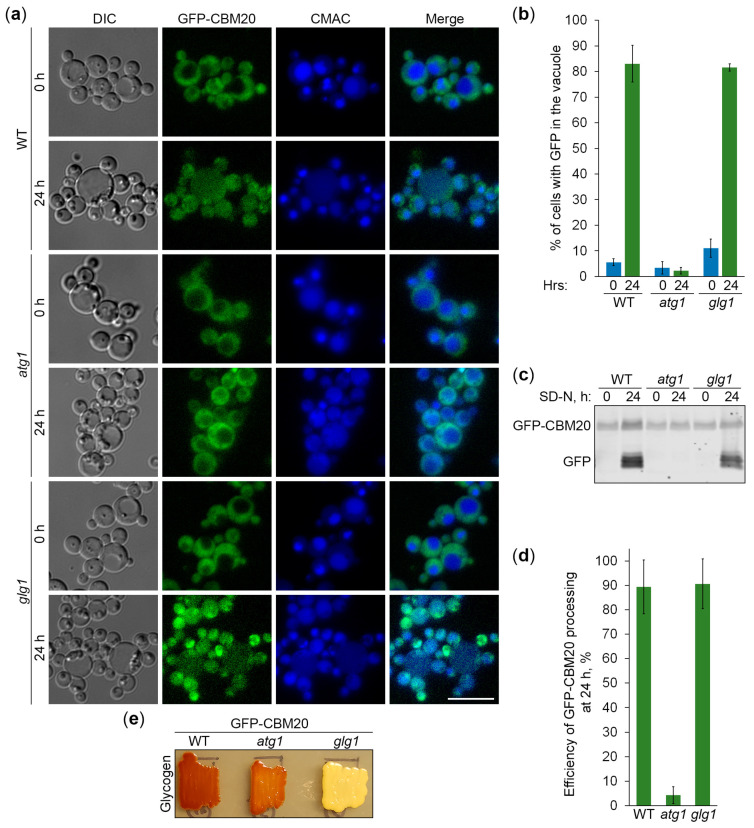
GFP-CBM20-marked glycogen is a neutral autophagic cargo in *K. phaffii*. (**a**) Delivery of GFP-CBM20 to the vacuole. WT, *atg1*, and *glg1* cells expressing GFP-CBM20 from *GSY1* promoter were grown in YPD for 1 day. A fraction of the cells was transferred to SD-N for 24 h. The rest of the YPD cultures were stained with CMAC for 30 min and imaged as “0 h”. The last 30 min of SD-N cultures was incubation with CMAC before imaging them as “24 h”. Scale bar, 10 µm. (**b**) Quantification of images in (**a**). Displayed are averages and standard deviations. (**c**) Processing of GFP-CBM20 in the vacuole. The same cells were grown in YPD for 1 day. A fraction of the cells was transferred to SD-N. At 0 and 24 h, equal volumes of cultures (not equal biomass) were taken from SD-N for immunoblotting. (**d**) Quantification of immunoblotting in (**c**). Displayed are averages and standard deviations. (**e**) Glycogen content. The same cells were grown on YPD plate for 2 days and exposed to the vapor of iodine crystals for glycogen staining.

**Figure 7:**
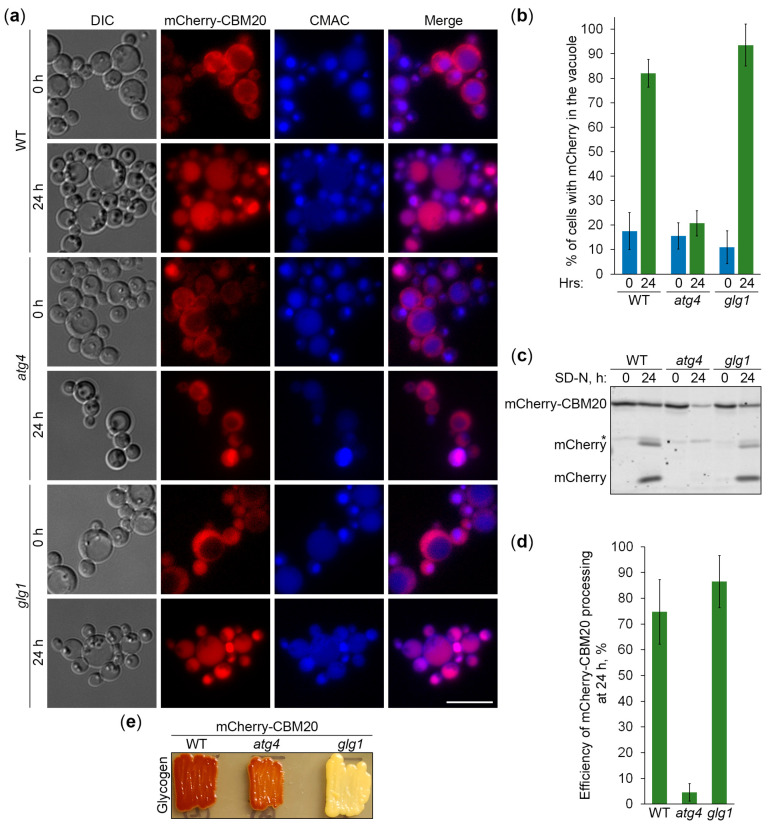
mCherry-CBM20-marked glycogen is a neutral autophagic cargo in *K. phaffii*. (**a**) Delivery of mCherry-CBM20 to the vacuole. WT, *atg4*, and *glg1* cells expressing mCherry-CBM20 from *GSY1* promoter were grown in YPD for 1 day. A fraction of the cells was transferred to SD-N for 24 h. The rest of the YPD cultures were stained with CMAC for 30 min and imaged as “0 h”. The last 30 min of SD-N cultures was incubation with CMAC before imaging them as “24 h”. Scale bar, 10 µm. (**b**) Quantification of images in (**a**). Displayed are averages and standard deviations. (**c**) Processing of mCherry-CBM20 in the vacuole. The same cells were grown in YPD for 1 day. A fraction of the cells was transferred to SD-N. At 0 and 24 h, equal volumes of cultures (not equal biomass) were taken from SD-N for immunoblotting. *, non-autophagic band. (**d**) Quantification of immunoblotting in (**c**). Displayed are averages and standard deviations. (**e**) Glycogen content. The same cells were grown on YPD plate for 2 days and exposed to the vapor of iodine crystals for glycogen staining.

Next, we used the constructed CBM20-based reporters of glycogen autophagy to address the question of whether *K. phaffii* GGs are its preferred, non-preferred, or neutral substrates. First, we compared the vacuolar delivery of GFP-CBM20 (Figure 6a) or mCherry-CBM20 (Figure 7a) reporters in WT and *glg1* cells using either *atg1* (Figure 6a) or *atg4* (Figure 7a) cells as a negative control. Quantification of epifluorescence microscopy results showed that the percentage of cells with GFP (Figure 6b) or mCherry (Figure 7b) signals inside the vacuole after 24 h of nitrogen starvation was not much different in *glg1* cells compared to WT cells. A higher percentage in *glg1* cells without GGs would mean that GGs are non-preferred substrates of bulk autophagy in WT cells, whereas a lower percentage in *glg1* cells would suggest that GGs are preferred substrates of autophagy in WT cells. However, the CBM20-based GG markers either on GGs (in WT cells) or in cytosol (in *glg1* cells) produced essentially the same vacuolar trafficking result, indicating that GGs, similar to cytosol, are a neutral cargo of bulk autophagy in *K. phaffii*.

To confirm the vacuolar delivery results above using vacuolar degradation data, we tested the GFP-CBM20 (Figure 6c) and mCherry-CBM20 (Figure 7c) processing in the same sets of strains by immunoblotting. The immunoblotting did not identify any big differences in the processing of CBM20-based reporters to GFP (Figure 6d) or mCherry (Figure 7d) between WT and *glg1* strains after 24 h of nitrogen starvation, strengthening our conclusion that glycogen is a neutral cargo of bulk autophagy in *K. phaffii*. Since there was no considerable difference in the processing of reporters between WT and *glg1* strains, we also compared the glycogen content in these strains with either GFP-CBM20 (Figure 6e) or mCherry-CBM20 (Figure 7e) that were used in the delivery and degradation assays above. As expected, WT, *atg1*, and *atg4* cells with CBM20-based reporters synthesized glycogen, whereas *glg1* cells with CBM20-based reporters did not. Summarizing, in contrast to *S. cerevisiae*, glycogen in *K. phaffii* is neither a preferred nor a non-preferred cargo of bulk autophagy. It is a neutral cargo of nitrogen starvation-induced autophagy.

## 3. Discussion

In this study, we clarified the cargo properties of *K. phaffii* GGs during autophagy induced by nitrogen starvation. Previous work on *K. phaffii* suggested that GGs are degraded by non-selective bulk autophagy [18]. However, a study in *S. cerevisiae* proposed that GGs are non-preferred substrates of bulk autophagy [20]. To resolve this discrepancy, which might have resulted from the use of different reporters to measure GG autophagy (Glg1-GFP in *K. phaffii* versus Gsy2-GFP in *S. cerevisiae*), we first identified *K. phaffii* Gsy1 as the only ortholog of the *S. cerevisiae* Gsy1 and Gsy2 paralogs (Figure S1). Then, we showed that *K. phaffii* Gsy1-GFP marks GGs, as Glg1-GFP, which is a *bona fide* GG marker due to its covalent linkage with glycogen (Figure 1). Moreover, we confirmed that Gsy1-GFP reports about GG autophagy (Figure 2). Then, by comparing the vacuolar delivery and degradation of Gsy1-GFP in strains with and without GGs, we concluded that GGs are neutral substrates of bulk autophagy in *K. phaffii*, because Gsy1-GFP either on GGs or in the cytosol was equally well delivered to the vacuole and degraded there (Figure 3).

To verify our findings with Gsy1-GFP, we created a new set of reporters based on the glycogen-binding CBM20 domain of human STBD1 protein, which acts as the autophagic receptor for GGs in mammalian cells [14,15]. For this, we fused CBM20 with either GFP or mCherry and placed these fusions under two different promoters, either a weaker *ATG8* promoter or a stronger *GSY1* promoter, for greater versatility (Figure 1). Then, we showed that GFP-CBM20 and mCherry-CBM20 fusions, like Glg1-GFP and Gsy1-GFP fusions, mark GGs in *K. phaffii* (Figure 1). Furthermore, we proved that these CBM20 fusions report about GG autophagy and can be used to validate Gsy1-GFP results (Figure 4 and Figure 5). The comparison of the delivery of CBM20 fusions to the vacuole and their degradation therein in cells with and without glycogen showed that glycogen does not affect autophagic flux of these glycogen-binding proteins, i.e., glycogen is neither a preferred nor a non-preferred cargo of bulk autophagy in *K. phaffii* (Figure 6 and Figure 7), in contrast to *S. cerevisiae* [20]. Instead, glycogen is a neutral cargo, like cytosol (where these reporters localize in the absence of glycogen).

Collectively, the studies in two models, *K. phaffii* and *S. cerevisiae*, indicate that GGs might have different fates during nitrogen starvation in different species. Despite the same pathway (bulk autophagy) being involved, in *K. phaffii*, GGs proceed to the vacuole and are degraded there, as cytosolic proteins (Figure 3, Figure 6 and Figure 7), and in *S. cerevisiae*, GGs are excluded from non-selective autophagic degradation till they can be selectively recruited to autophagosomes by the autophagic receptor Atg45 [20]. Consequently, the autophagy of GGs in *S. cerevisiae* is postponed until prolonged starvation. While it is clear how *S. cerevisiae* cells ramp up GG autophagy during prolonged starvation (via increased expression of Atg45 [20]), it is unclear how they exclude GGs from autophagosomes before that. Such a negative regulation of cargo sequestration by bulk autophagy is new and worth further studies. Further studies are also necessary to clarify evolutionary differences between the two yeast species regarding the glycophagy receptor, Atg45. While some autophagic receptors, such as Atg32 [26], are conserved in *K. phaffii*, others, such as Atg19 [27], are not. Yet some receptors, like Atg30 in *K. phaffii* [28] and Atg36 in *S. cerevisiae* [29], are functional counterparts. The study on *S. cerevisiae* Atg45 suggested that it is not conserved in other yeasts beyond the *Saccharomycetaceae* family and *Candida albicans* [20]. However, this does not exclude the possibility that a functional (if not structural) ortholog of *S. cerevisiae* Atg45 might be present in some (if not all) yeast species.

The studies in yeasts have also set a stage for future studies in mouse models, which are necessary to clarify if GGs are neutral or preferred substrates during STBD1-independent autophagy of GGs in skeletal and cardiac muscles [16,17]. While glycogen is clearly a preferred cargo of STBD1-dependent glycophagy in mouse liver [17], its cargo properties in non-hepatic mammalian tissues remain unknown. Also, it is not entirely clear why autophagy of GGs is STBD1-independent there. Since the expression of STBD1 in the heart is much lower than that in the liver of Pompe disease mice, it could explain this discrepancy for the heart [16]. However, the expression of STBD1 in skeletal muscles is even higher than that in the liver, but knockdown [16] or knockout [17] of *STBD1* in skeletal muscles of Pompe disease mice does not affect their lysosomal glycogen accumulation. Therefore, either another autophagic receptor plays a major role in glycophagy in skeletal and cardiac muscles (if GGs are preferred substrates) or glycogen autophagy proceeds non-selectively there (if GGs are neutral substrates, like in *K. phaffii*).

## 4. Materials and Methods

### 4.1. Strains and Plasmids

Table 1 describes the *K. phaffii* strains and plasmids that were used in this study. After cloning, all the polymerase chain reaction (PCR) fragments were verified by sequencing. The *K. phaffii* recipient strains were transformed with plasmids by electroporation [30]. Before transformation, plasmids were linearized with the endonucleases of restriction (see below for details) for efficient yeast genome integration.

Generation of the integrative plasmid, pNW10, with the P*_GLG1_*-*PGK1-GFP* expression cassette was described before [18]. The integrative plasmid, pRK23, with the P*_GSY1_*-*GSY1-GFP* expression cassette (Figure 1a) has the 293 bp *GSY1* promoter and *GSY1* open reading frame (ORF) without a STOP codon. They were PCR amplified and cloned as a single XmaI-PstI fragment into the vector pRK1 [31]. We also created 4 integrative plasmids, pRK29, pRK34, pRK28, and pRK32, with P*_ATG8_*-*GFP-CBM20,* P*_GSY1_*-*GFP-CBM20*, P*_ATG8_*-*mCherry-CBM20*, and P*_GSY1_*-*mCherry-CBM20* expression cassettes, respectively (Figure 1a). The mCherry-CBM20 plasmids were built as follows. pRK28 was constructed by replacing the *K. phaffii ATG8* ORF as an AsiSI-HindIII fragment on pJCF477 [32] with an AsiSI-HindIII fragment containing the nucleotide sequence of human STBD1′s CBM20 domain with a STOP codon, as on GYSC [23]. pRK32 was made by replacing the *K. phaffii ATG8* promoter as an XmaI–XmaI fragment on pRK28 with the XmaI–XmaI fragment containing the PCR amplified 293 bp *GSY1* promoter. The GFP-CBM20 plasmids were generated as follows. pRK29 was built by replacing the *K. phaffii ATG8* ORF as an AsiSI-SpeI fragment on pJCF760 (a gift from Jean-Claude Farré) with the AsiSI-SpeI fragment containing the nucleotide sequence of human STBD1′s CBM20 domain with a STOP codon, as on GYSC [23]. pRK34 was made in two steps. First, we created pRK33 by cloning the 293 bp *GSY1* promoter as an XmaI–XmaI fragment from pRK32 into the vector, pIB1 [33]. Then, we built pRK34 by inserting the SpeI–SpeI fragment containing the PCR amplified *GFP-CBM20* ORF (from pRK29) into the pRK33 vector.

Before transformation, all plasmids with the *HIS4* selectable marker (pNW10, pRK23, pRK29, and pRK34) were linearized in *HIS4* using EcoNI for their integration into the *his4* locus of recipient cells. His^+^-transformants were selected on SD+DOM-His plates [18] and screened for the expression of GFP fusions by immunoblotting (see Section 4.2). Likewise, plasmids with the *ARG4* selectable marker, pRK28 and pRK32, were linearized in *ARG4* using NruI and BglII, respectively, for their integration into the *arg4* locus of recipient cells. Arg^+^-transformants were selected on SD+DOM-Arg plates (1.7 g/L yeast nitrogen base [YNB] without amino acids and ammonium sulfate, 5 g/L ammonium sulfate, 1.92 g/L drop-out mix synthetic minus arginine, 20 g/L dextrose, and 20 g/L agar) and screened for the expression of mCherry fusions by immunoblotting (see Section 4.2).

**Table 1:** *K. phaffii* strains and plasmids that were used in this study.

Mutant	Strain	Background	Genotype and Plasmid	Source
WT	PPY12h	PPY12h	*arg4 his4*	[34]
WT	SRK147	PPY12h	*his4*::pRK22 *(*P*_GLG1_-GLG1-GFP, HIS4)*	[18]
WT	SNW78	PPY12h	*his4*::pNW10 *(*P*_GLG1_-PGK1-GFP, HIS4)*	This study
WT	SRK152	PPY12h	*his4*::pRK23 *(*P*_GSY1_-GSY1-GFP, HIS4)*	This study
WT	SRK176	PPY12h	*his4*::pRK29 *(*P*_ATG8_-GFP-CBM20, HIS4)*	This study
WT	SRK213	PPY12h	*his4*::pRK34 *(*P*_GSY1_-GFP-CBM20, HIS4)*	This study
WT	SNW57	PPY12h	*arg4*::pRK28 *(*P*_ATG8_-mCherry-CBM20, ARG4)*	This study
WT	SNW59	PPY12h	*arg4*::pRK32 *(*P*_GSY1_-mCherry-CBM20, ARG4)*	This study
*atg1*	R12	GS115	*atg1-1*::*Zeocin^R^ his4*	[21]
*atg1*	SRK154	R12	*his4*::pRK23 *(*P*_GSY1_-GSY1-GFP, HIS4)*	This study
*atg1*	SRK178	R12	*his4*::pRK29 *(*P*_ATG8_-GFP-CBM20, HIS4)*	This study
*atg1*	SRK215	R12	*his4*::pRK34 *(*P*_GSY1_-GFP-CBM20, HIS4)*	This study
*atg4*	PPM408	PPY12h	*atg4*::*Zeocin^R^ arg4 his4*	[24]
*atg4*	SNW55	PPM408	*arg4*::pRK28 *(*P*_ATG8_-mCherry-CBM20, ARG4)*	This study
*atg4*	SNW62	PPM408	*arg4*::pRK32 *(*P*_GSY1_-mCherry-CBM20, ARG4)*	This study
*prA,B*	SMD1163	GS115	*pep4 prb1 his4*	[22]
*prA,B*	SRK157	SMD1163	*his4*::pRK23 *(*P*_GSY1_-GSY1-GFP, HIS4)*	This study
*prA,B*	SRK180	SMD1163	*his4*::pRK29 *(*P*_ATG8_-GFP-CBM20, HIS4)*	This study
*prA,B*	SRK217	SMD1163	*his4*::pRK34 *(*P*_GSY1_-GFP-CBM20, HIS4)*	This study
*ypt7*	SRRM197	PPY12h	Δ*ypt7*::*Geneticin^R^ arg4 his4*	[25]
*ypt7*	SPB1	SRRM197	*arg4*::pRK28 *(*P*_ATG8_-mCherry-CBM20, ARG4)*	This study
*ypt7*	SPB2	SRRM197	*arg4*::pRK32 *(*P*_GSY1_-mCherry-CBM20, ARG4)*	This study
*glg1*	SNW49	PPY12h	Δ*glg1::Zeocin^R^* (pNW9)	[18]
*glg1*	SNW51	SNW49	*his4*::pRK23 *(*P*_GSY1_-GSY1-GFP, HIS4)*	This study
*glg1*	SNW53	SNW49	*his4*::pRK34 *(*P*_GSY1_-GFP-CBM20, HIS4)*	This study
*glg1*	SNW64	SNW49	*arg4*::pRK32 *(*P*_GSY1_-mCherry-CBM20, ARG4)*	This study

### 4.2. Immunoblotting

For immunoblotting, *K. phaffii* cells were processed as before [18]. In brief, they were grown in 1 mL of YPD medium for 1 day at 30 °C. Then, 3 ODs of cells were washed twice with 1 mL of 1× YNB without amino acids and ammonium sulfate and resuspended in 3 mL of SD-N medium (starting OD_600_ = 1) to study the nitrogen starvation-induced autophagy. For this, 1 mL of culture was taken at 0 and 24 h from SD-N. Protein lysates were prepared by trichloroacetic acid precipitation [35] and assayed by immunoblotting with anti-GFP (11814460001, Roche Diagnostics, Mannheim, Germany) or anti-mCherry (PA5-34974, Invitrogen, Carlsbad, CA, USA) antibodies. Nitrocellulose membranes were imaged on the Odyssey CLx imager (LI-COR Biosciences, Lincoln, NE, USA) and the images were quantified in the LI-COR Image Studio Lite v5.2 software. All immunoblotting experiments were performed three times in duplicate.

### 4.3. Epifluorescence Microscopy

For epifluorescence microscopy, *K. phaffii* cells were processed as before [18]. Briefly, they were grown in YPD, washed, and transferred to SD-N, as above. Then, the remaining YPD cultures were stained with the CellTracker blue CMAC dye (C2110, Invitrogen, Eugene, OR, USA) for 30 min at 30 °C and imaged as a “0 h” or “0 min” time-point. The last 30 min of SD-N cultures was incubation with CMAC dye before imaging them as a “24 h” or “30 min” time-point. For imaging, cells were immobilized in 1% low-melt agarose, as previously described [18]. Cells in 5 non-overlapping fields of view were imaged for each strain at each time-point on the Eclipse Ti2-E inverted microscope operated by the NIS Elements AR v5.20 software (Nikon Instruments Inc., Melville, NY, USA). All epifluorescence microscopy experiments were performed at least twice in duplicate.

### 4.4. Confocal Microscopy

For confocal microscopy, *K. phaffii* cells were grown in YPD, as above. For imaging, cells were immobilized in 1% low-melt agarose, as previously described [18]. Cells in 3 non-overlapping fields of view were imaged for each strain on the Zeiss LSM 980 confocal microscope with Airyscan 2 (Carl Zeiss Microscopy LLC, White Plains, NY, USA) using the Airyscan Superresolution mode. The 488 and 561 nm lasers were used for excitation of GFP and mCherry, respectively. Z-stack images were captured with an interval of 0.2 μm. Images were processed using the ZEN lite 3.9 software (Carl Zeiss Microscopy LLC, White Plains, NY, USA).

### 4.5. Glycogen Staining

For glycogen staining, *K. phaffii* cells were processed as before [18]. Briefly, patches of biomass were grown on YPD plates for 2 days at 30 °C. Then, the plates were inverted over crystals of iodine for 2 min. The patches with glycogen turned brown.

### 4.6. Statistical Analysis

Microsoft Excel 2016, version 16.87 (24071426), software was used for statistical analysis of the data obtained from at least two independent experiments in duplicate (N ≥ 4). The results are displayed as average ± standard deviation. Statistical significance was probed with Student’s t-test (two-tailed distribution, two-sample unequal variance). Differences between two sample groups were considered statistically significant if *p* < 0.05.

## 5. Conclusions

In this study, we developed new experimental tools that helped us to shed light on interspecies differences in the autophagy of GGs. Precisely, using glycogen synthase- and CBM20-based reporters of GG autophagy in *K. phaffii* strains with and without glycogen, we revealed that *K. phaffii* GGs do not affect the autophagic flux of these reporters and, thus, constitute neutral substrates of bulk autophagy, in contrast to *S. cerevisiae* where GGs are non-preferred substrates of bulk autophagy. As such, our study sets the stage for future studies on GG autophagy in mammals and offers new tools to explore this process.

## Data Availability

The original contributions presented in the study are included in the article, further inquiries can be directed to the corresponding author.

## Supplementary Materials

The following supporting information can be downloaded at: https://www.mdpi.com/article/10.3390/ijms252111772/s1. Reference [36] is cited in Figure S1 legend.
