# Supplementary material for: New Toolset of Reporters Reveals That Glycogen Granules Are Neutral Substrates of Bulk Autophagy in Komagataella phaffii
Source: Int J Mol Sci. 2024 Nov 1;25(21):11772. doi: 10.3390/ijms252111772 (PMC11546884; doi:10.3390/ijms252111772)
Supplement: Supplementary file 1 [file ijms-25-11772-s001.zip › ijms-3255383-supplementary.pdf]

```
           1        10        20        30        40        50        60        70        80        90        100       110       120       130
ScGsy1     MARDLQNHLLFEVATEVTNRVGGIYSVLKSKAPVTVAQYGDNYTLLGPLNKATYESEVEKLDWEDESIFPEELLPIQKTLMSMREKGVNFVYGNWLIEGAPRVILFELDSVRHFLNEWKADLWSLVGIPS
ScGsy2     MSRDLQNHLLFETATEVANRVGGIYSVLKSKAPITVAQYKDHYHLIGPLNKATYQNEVDILDWKKPEAFSDEMRPVQHALQTMESRGVHFVYGRWLIEGAPKVILFDLDSVRGYSNEWKGDLWSLVGIPS
KpGsy1     MGKDITNHLLFEIATEVANRVGGIYSVLKSKAPVTVAEYKDRYTLIGPLNANSANVEVEELPVLDPV--------LKRTLDSMSGRGIRYLYGRWLIEGAPRVILFDIGSAAGYLNEWKSDLWNIAGVPT
Consensus  M.rDlqNHLLFE.ATEVaNRVGGIYSVLKSKAP!TVA#YkD.YtLiGPLNkaty#.EV#.Ldw.dp..f..e..p.q.tL.sM..rG!.%vYGrWLIEGAPrVILF#ldSvrg%lNEWK.DLWslvG!Ps

           131     140       150       160       170       180       190       200       210       220       230       240       250       260
ScGsy1     PEHDHETNDAILLGYVVVWFLGEVSKLDS-SHAIIGHFHEWLAGVALPLCRKKRIDVVTIFTTHATLLGRYLCAAGDVDFYNNLQYFDVDQEAGKRGIYHRYCIERAAAHTADVFTTVSQITALEAEHLL
ScGsy2     PENDFETNDAILLGYTVAWFLGEVAHLDS-QHAIVAHFHEWLAGVALPLCRKRRIDVVTIFTTHATLLGRYLCASGSFDFYNCLESVDVDHEAGRFGIYHRYCIERAAAHSADVFTTVSQITAFEAEHLL
KpGsy1     PEHDVETNDAILLGYLVAWFLGELVHNDGHKRAIICHAHEWLAAVCLPLCRKRKIDVTTVFTTHATLLGRYLCA-GSVDFYNNLDKFDVDAEAGKRGIYHRYCIERAAAHSADVFTTVSHITAYEAEHLL
Consensus  PEhD.ETNDAILLGY.VaWFLGEv.hlDs..hAI!.HfHEWLAgVaLPLCRKrrIDVvT!FTTHATLLGRYLCA.GsvDFYNnL#.fDVD.EAGkrGIYHRYCIERAAAHsADVFTTVSqITA.EAEHLL

           261     270       280       290       300       310       320       330       340       350       360       370       380       390
ScGsy1     KRKPDGILPNGLNVVKFQAVHEFQNLHALKKDKINDFVRGHFHGCFDFDLDNTVYFFIAGRYEYKNKGADMFIESLARLNYRLKVSGSKKTVVAFLIMPAKTNSFTVEALKSQAIVKSLENTVNEVTASI
ScGsy2     KRKPDGILPNGLNVIKFQAFHEFQNLHALKKEKINDFVRGHFHGCFDFDLDNTLYFFIAGRYEYKNKGADMFIEALARLNYRLKVSGSKKTVVAFIVMPAKNNSFTVEALKGQAEVRALENTVHEVTTSI
KpGsy1     KRKPDGVLPNGLNVIKFQAVHEFQNLHAEKKEKINEFIRGHFYGQLDFKLNNTLYFFIAGRYEYRNKGVDFFIESLARLNHRLKASNSNMTVVAFIIMPAKTNSYTVETLKGQAVIKQLENTINDIEALV
Consensus  KRKPDG!LPNGLNV!KFQAvHEFQNLHAlKK#KIN#F!RGHFhGcfDFdL#NTlYFFIAGRYEYkNKGaDmFIEsLARLNyRLKvSgSkkTVVAFi!MPAKtNS%TVEaLKgQA.!k.LENT!n#!tas!

           391     400       410       420       430       440       450       460       470       480       490       500       510       520
ScGsy1     GKRIFEHTMRYPHNGLESELPT-NLDELLKSSEKVLLKKRVLALRRPYGELPPVVTHNMCDDANDPILNQIRHVRLFNDSSDRVKVIFHPEFLNANNPILGLDYDEFVRGCHLGVFPSYYEPWGYTPAEC
ScGsy2     GKRIFDHAIRYPHNGLTTELPT-DLGELLKSSDKVMLKRRILALRRPEGQLPPIVTHNMVDDANDLILNKIRQVQLFNSPSDRVKMIFHPEFLNANNPILGLDYDEFVRGCHLGVFPSYYEPWGYTPAEC
KpGsy1     GKRLFEFCQHPATSSNSSHIEMPDIDDLLKPSDRVLLKRRMFAMKRE--GLPPIVTHNMADDSTDPILNQIRNVQLFNRPEDRVKIIFHPEFLNSNNPILSLDYDEFVRGCHLGVFPSYYEPWGYTPAEC
Consensus  GKRiF#h..ryphngl.selpt.#ld#LLKsS#kV$LKrR.lA$rRp.g.LPP!VTHNM.DDanDpILNqIR.VqLFN.psDRVK.IFHPEFLNaNNPILgLDYDEFVRGCHLGVFPSYYEPWGYTPAEC

           521     530       540       550       560       570       580       590       600       610       620       630       640       650
ScGsy1     TVMGVPSITTNVSGFGAYMEDLIETDQAKDYGIYIVDRRFKSPDESVEQLADYMEEFVNKTRRQRINQRNRTERLSDLLDWKRMGLEYVKARQLGLRRAYPEQFKQLVGETISDANMNTLAGGKKFKIAR
ScGsy2     TVMGVPSITTNVSGFGAYMEDLIETNQAKDYGIYIVDRRFKAPDESVEQLVDYMEEFVKKTRRQRINQRNRTERLSDLLDWKRMGLEYVKARQLALRRGYPDQFRELVGEELNDSNMDALAGGKKLKVAR
KpGsy1     TVMGIPSITTNLSGFGCYMEDLIEN--SSDYGIYIVDRRLKSVDESIDQLTSYMFDFVDKTRRQRINQRNRVERLSDLLDWRRMGLEYVKGRSLALRRAYPDEFKNQ-----NNEHSNPFSESNSIKITR
Consensus  TVMG!PSITTNvSGFGaYMEDLIEt.qakDYGIYIVDRRfKspDES!#QL.dYMe#FV.KTRRQRINQRNRtERLSDLLDWkRMGLEYVKaRqLaLRRaYP##Fk#lvge..n#.nm#.laggkk.K!aR

           651     660       670       680       690       700       710       721
ScGsy1     PLSVPGSPK-VRSNSTVYMTPGDLGTLQDANNADDYFNLSTNGAIDNDDDDNDTSAYYEDN
ScGsy2     PLSVPGSPRDLRSNSTVYMTPGDLGTLQEVNNADDYFSLGVNPAADDDDD----GPYADDS
KpGsy1     PLSVPGSPRDKYSSSANIMTPGDLGTLQENNSTDYISWRLGNDTEEDGLDVTLRGLSVAPSAEQSGDEFDA
Consensus  PLSVPGSPrd.rSnStvyMTPGDLGTLQ#.NnaDdyf.l..N.a.##ddD....g.y..ds..........
```

Figure S1. Multiple sequence alignment of *K. phaffii* Gsy1 and its orthologues in *S. cerevisiae*. ScGsy1, *S. cerevisiae* glycogen synthase 1 (YFR015C); ScGsy2, *S. cerevisiae* glycogen synthase 2 (YLR258W); KpGsy1, *K. phaffii* glycogen synthase 1 (XP_002493014.1). Color code of amino acid residues: red – high consensus, blue – low consensus, black – neutral. The alignment was generated using MultAlin software [36]. Alignment parameters: Blosum62 – 12 – 2 (symbol comparison table – gap penalty at opening – gap penalty at extension).
